# Identification of Key Predictors of Cryoglobulinemia Severity at Diagnosis: Threshold, Type, and Severity Score at Diagnosis

**DOI:** 10.3390/jcm14020556

**Published:** 2025-01-16

**Authors:** Jerome Razanamahery, Nils Aubertin, Maxime Bach Bunner, Gilles Blaison, Bastien Bouldoires, Thibaud Soumagne

**Affiliations:** 1 Department of Internal Medicine and Clinical Immunology, Dijon University Hospital, 21000 Dijon, France; 2 Department of Geriatric Medicine, Centre Départementale de Repos et de Soins, 68026 Colmar, France; nils.aubertin@ch-colmar.fr; 3 Department of Internal Medicine and Clinical Immunology, Hopitaux Civils de Colmar, 68026 Colmar, France; maxime.bachbunner@ch-colmar.fr (M.B.B.); giilles.blaison@ch-colmar.fr (G.B.); 4 Department of Internal Medicine and Clinical Immunology, Hopital de Macon, 71000 Macon, France; babouldoires@ch-macon.fr; 5 Department of Pulmonary Medicine, European Hospital Georges Pompidou, 75015 Paris, France; thibaud.soumagne@aphp.fr

**Keywords:** cryoglobulinemia, vasculitis, severity, score, diagnosis methods

## Abstract

**Background**: Cryoglobulinemia (CG) syndrome is a heterogeneous condition characterized by the presence of cryoglobulins in serum, often leading to vasculitis with protean clinical manifestations. Understanding the presentation of cryoglobulinemia-related symptoms based on cryoprecipitate levels, GC type, and severity at diagnosis is essential for effective management. Hence, this study aimed to provide a comprehensive analysis of patients with positive cryoglobulin detection to investigate these aspects. **Methods**: We conducted a retrospective review of clinical charts from patients with positive cryoglobulin detection at Colmar Hospital between May 2015 and April 2019. **Results**: Among 166 patients with positive cryoglobulins, the median cryoprecipitate value was 37 mg/L [IQR: 25–70], with 62% of patients below the 50 mg/L threshold. High cryoprecipitate levels were associated with C-virus hepatitis (*p* = 0.0007), increased fatigue (*p* = 0.001), fever (*p* = 0.0013), weight loss (*p* = 0.028), and musculoskeletal symptoms (*p* = 0.002). These patients also exhibited decreases in complement fractions (*p*-values 0.017 to 0.006). At the end of the one-year follow-up, they required frequent renal replacement therapy (*p* < 0.0001) and had a higher mortality rate (*p* = 0.02). Based on the CG type, patients with type I GC had splenomegaly (*p* = 0.039) and hemopathy (*p* = 0.001). According to severity at initial presentation, the severe patients had more purpura (*p* < 0.001), Raynaud’s phenomenon (*p* = 0.039), and leukocytoclastic vasculitis on skin biopsy (*p* < 0.001), along with higher cryoprecipitate levels (*p* = 0.011). Multivariate analysis identified purpura (OR: 10.25), hematological malignancy (OR: 7.06), Raynaud’s phenomenon (OR: 6.41), and cryoprecipitate levels (OR: 1.02) as significant markers of disease severity serving for the development of a severity score for clinical practice. **Conclusions**: This study identifies severity markers in patients with positive cryoprecipitate and proposes a score related to severity at diagnosis.

## 1. Introduction

Cryoglobulinemia (CG) encompasses a spectrum of heterogeneous conditions characterized by the presence of cryoglobulins in serum [1]. These immunoglobulins (Ig) precipitate below 37 °C in vitro and redissolve upon rewarming [2,3]. CG is classified into types based on the Ig isotype [2], distinguishing type I CG (associated with hematological conditions) from mixed CG (types II and III) [4,5]. Patients experience diverse symptoms, including constitutional, musculoskeletal, and vascular manifestations, which define the spectrum of CG vasculitis [6,7]. While cryoglobulins may sometimes be detected incidentally, some patients present with severe, life-threatening manifestations [8]. Factors influencing disease severity include the type of cryoglobulin (type I or mixed), cryoprecipitate levels, the presence of underlying conditions (e.g., hematological malignancy, autoimmune disorders, chronic infection), and multi-organ involvement [9,10,11]. Despite sharing a common name, type I and mixed CG differ in pathophysiological mechanisms and prognosis CG [4,10].

Cryoprecipitate levels exceeding 50 mg/L are considered clinically significant [12,13,14], but patients with lower levels can also experience severe, life-threatening complications [15,16].

Treatment strategies are tailored according to disease severity, CG type, and the presence of underlying conditions. These approaches include observation, steroid therapy with or without plasmapheresis, and immunosuppressive agents [17,18,19]. Due to delays in identifying cryoglobulins, treatment decisions are often guided by clinical judgment and expert guidelines [1,8,20].

Given these challenges, this study aims to investigate the characteristics of CG patients based on cryoprecipitate levels and CG type and to identify factors associated with disease severity at the time of diagnosis.

## 2. Methods

### 2.1. Study Design and Population

We conducted a retrospective review of clinical charts from patients with positive cryoglobulin detection at Colmar Hospital between May 2015 and April 2019. Patients with cryoglobulins present, via laboratory testing [21] in the setting of compatible clinical symptoms, were included. These symptoms included weight loss, arthralgia, vascular manifestations (e.g., skin necrosis or Raynaud’s phenomenon), neuropathy, and glomerulopathy [7].

### 2.2. Investigations

Cryoprecipitate detection was performed using spectrometry combined with immunofixation to determine Ig-type after 7 days of storage [21]. All patients underwent screening for chronic infectious diseases (human immunodeficiency virus, hepatitis B and C), autoimmune-related disorders (antinuclear antibody and anti-CCP testing), and malignancy (blood immunophenotyping and body computed tomography).

Skin biopsies were performed selectively in patients who presented suspicious cutaneous lesions (e.g., palpable purpura, necrotic ulcers).

Electromyograms (EMG) were only performed in patients with clinical suspicion of neuropathy (e.g., persistent sensory deficits, tingling, numbness, or motor weakness). Compatible neurologic features on EMG referred to findings consistent with peripheral neuropathy (e.g., sensory axonal neuropathy), as interpreted by the neurology team.

### 2.3. Exclusion Criteria

Patients with vasculitis not clearly attributable to cryoglobulins (e.g., ANCA-associated vasculitis, IgA vasculitis, or middle or large vessel vasculitis) were excluded.

### 2.4. Data Collection

Collected variables analyzed included demographics, medical history, clinical symptoms, and laboratory results. Treatment regimens varied and included steroids alone, Ig-IV, plasmapheresis, conventional immunosuppressive agents, anti-CD20 therapy, chemotherapy, and combined therapy (defined by the use of at least two agents).

### 2.5. Outcomes

Outcomes analyzed at one year post-diagnosis included relapse-free survival, progression toward chronic disease (defined by at least 2 relapses), end-stage renal failure requiring replacement therapy, and mortality.

### 2.6. Study Analysis

1: Cryoprecipitate levels: Patients were stratified by cryoprecipitate levels at initial measurement, with levels above 50 mg/L considered high [14].

2: CG type: Patients were compared based on CG type (type I vs. mixed).

3: Severity: Patients were classified according to disease severity at the initial evaluation. Severity was defined as the initiation of steroids or a >50% increase in the daily dose or initiation of other therapies within three days of diagnosis, based on the physician’s discretion. Further severity analysis included items from the Birmingham vasculitis activity score (BVAS) 2003.

### 2.7. Statistical Analysis

Quantitative data are presented as medians with interquartile ranges [IQR] and compared using Mann–Whitney tests. Qualitative data, expressed as numbers (percentages), were compared using Chi-squared or Fisher’s exact tests.

To identify factors associated with severe disease, we performed logistic regression analysis with univariate and multivariate models. A predictive score for disease severity was developed using variables with a *p*-value ≤ 0.20 in the bivariate analysis, followed by stepwise selection. The final score included variables selected through a stepwise procedure and derived from the coefficients in the multivariate analysis. The optimal threshold for severe cryoglobulinemia syndrome was determined by maximizing sensitivity and specificity using the Youden index of receiver operating characteristic (ROC) curve analysis. Statistical significance was set at *p*-value < 0.05 (two-sided). Statistical analyses were performed using Prism 10 (GraphPad, San Diego, CA, USA), R version 4.0.3, and RStudio version 1.4.1103 (R Foundation for Statistical Computing, Vienna, Austria).

The study was approved by the ethics committee of Colmar Hospital and followed the principles of the Declaration of Helsinki.

## 3. Results

Among the 166 patients with the presence of cryoglobulins in serum, the median cryoglobulin level was 37 mg/L [IQR: 25–70].

Comparison according to cryoprecipitate levels:

A total of 62% (102/166) of patients exhibited cryoprecipitate levels below 50 mg/L (median: 27 mg/L [IQR: 22–33]), whereas 38% (64/166) had levels above 50 mg/L (median: 90 mg/L [60–157]). The distribution of cryoglobulinemia types (type I and mixed) was similar between the two groups. Patients with high cryoprecipitate levels have more frequently C-virus hepatitis (7/102 (6.8%) vs. 17/64 (26%); *p* = 0.0007), when other baseline conditions were similarly distributed. 

Constitutional symptoms, including fatigue (17/64 (27%) vs. 6/102 (5%); *p* = 0.001), fever (11/64 (17%) vs. 5/102 (4%); *p* = 0.013), and weight loss (6/64 (9%) vs. 0/102 (0%); *p* = 0.028), were significantly more prevalent in patients with high cryoprecipitate levels. Musculoskeletal symptoms were also more frequent in this group (11/64 (17%) vs. 3/102 (2%); *p* = 0.002), while vascular symptoms (e.g., purpura, Raynaud’s phenomenon, livedo, necrosis, or vasculitis on biopsy) were similarly distributed. Patients with high cryoprecipitate levels exhibited lower complement fractions (CH 50: 43 [20–61] vs. 64 [46–76]; *p* = 0.017; C3: 0.9 [0.65–1.19] vs. 1.08 [0.82–1.35]; *p* = 0.028; C4: 0.12 [0.045–0.20] vs. 0.1850 [0.110–0.277]; *p* = 0.006) compared to those with low cryoprecipitate levels. Treatment strategies were similar according to cryoprecipitate levels.

During the one-year follow-up, patients with low cryoprecipitate levels were more likely to experience chronic disease evolution (>2 relapses) (63/102 (61%) vs. 0/64 (0%); *p* < 0.0001). In contrast, patients with high cryoprecipitate experienced fewer relapses (32/64 (50%) vs. 28/102 (27%); *p* = 0.0046) and required renal replacement therapy more frequently (12/64 (18%) vs. 1/102 (0.98%); *p* < 0.0001) (see Table 1).

Comparison according to cryoglobulinemia type:

When stratified by cryoglobulin type (type I (n = 16) vs. mixed CG patients (n = 150)), type I cryoglobulinemia was associated with a higher prevalence of malignancy (5/16 (31%) vs. 9/150 (6%); *p* = 0.0068), especially hematological conditions (8/16 (50%) vs. 18/150 (12%); *p* = 0.001), mainly Waldenström macroglobulinemia (7/16 (43%) vs. 0/150 (0%); *p* < 0.0001). C-virus hepatitis history tends to be more common in mixed GC (27/150 (18%) vs. 0/16 (0%); *p* = 0.07). Clinical features were similar except for frequent splenomegaly in type I CG (5/16 (31%) vs. 7/150 (4.6%); *p* = 0.039) and a trend toward more frequent musculoskeletal symptoms in mixed CG (25/150 (16.6%) vs. 0/16 (0%); *p* = 0.07). Patients with mixed cryoglobulinemia exhibited lower CH50 levels (median 51 UI/mL [32.25–76] vs. 78 [58–193]; *p* = 0.0350) (see Table 2). Patients with type I CG required a frequent combined therapeutic approach (6/16 (37%) vs. 22/150 (14%); *p* = 0.04), resulting in similar outcomes at one year.

Comparison according to severity at initial diagnosis.

Thus, patients were stratified based on severity at diagnosis, with 20% (34/166) categorized as severe.

Severe cryoglobulinemia syndrome was associated with more purpura (14/33 (42%) vs. 15/133 (11%); *p* < 0.001), Raynaud’s phenomenon (8/33 (26%) vs. 14/133 (10%); *p* = 0.039), and leukocytoclastic vasculitis on biopsy (10/15 (66%) vs. 4/26 (15%); *p* < 0.001). Severe patients had frequent type I CG (6/33 (18%) vs. 10/133 (7%); *p* = 0.048) (and all of them had IgM cryoprecipitate), secondary to hematological malignancies (9/33 (27%) vs. 17/133 (12%); *p* = 0.02).

**Figure 1:**
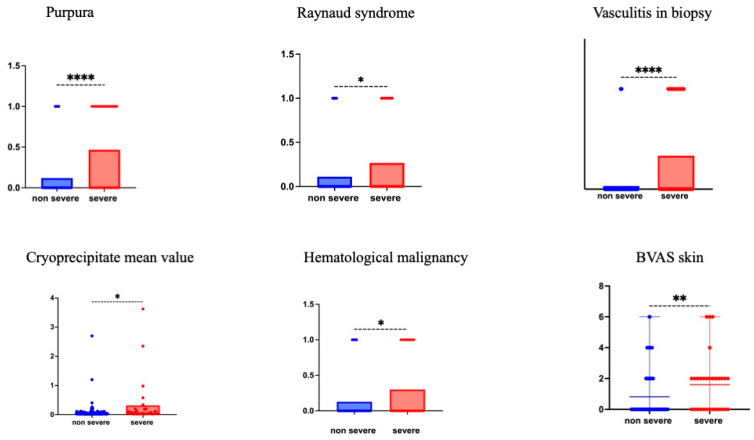
Patients characteristics according to CG severity at diagnosis. The items purpura, Raynaud’s phenomenon., vasculitis in biopsy, cryoprecipitate value, and hematological malignancy are presented by their mean values for more visibility. The skin items of the 2003 BVAS score are presented as scattered plot means with range. *p* value is the result of the Mann-Whitney test. * *p* < 0.05,** *p* < 0.01, **** *p* < 0.0001.

Biological differences included a lower white cell count (median [IQR] g/L: 5.8 [4–7.8] vs. 7.0 [5.1–9.4]; *p* = 0.036) and higher cryoprecipitate level in severe patients (median [IQR] mg/L: 54 [28–191] vs. 34 [25–65]; *p* = 0.011) (see Figure 1 and Table 3). Proteinuria > 0.5 g/day and hematuria (when available) were more frequent in non-severe patients (4/30 (13.3%) vs. 20/53 (37%) and (16.6%) vs. 23/52 (44%); *p* = 0.0234 and *p* = 0.0152, respectively).

These severe patients had a median BVAS [IQR] of 10 [8–12], whereas non-severe patients had a median BVAS [IQR] of 2 [0–4].

Treatment strategies differed slightly, with severe patients more frequently receiving steroids alone within the first three days (15% vs. 4%, *p* = 0.03). Outcomes at one year were similar across severity groups.

In the overall cohort, 18 patients were dead at one-year follow-up. Mortality was significantly higher in patients with elevated cryoprecipitate levels (11/64 (17.1%) vs. 7/102 (7%)) (*p* = 0.02). However, no significant associations were found between mortality and CG type (*p* = 0.47) or initial disease severity (*p* = 0.7).

Finally, multivariate analysis showed that factors associated with severity at diagnosis included the presence of purpura (odds ratio (OR): 10.25, CI95% [3.6231–59], *p* = 0.001), hematological conditions (OR: 7.06, CI95% [1.6329–49]; *p* = 0.007), Raynaud’s phenomenon (OR: 6.41, CI95% [1.9321–99]; *p* = 0.002), and elevated cryoprecipitate levels (OR: 1.02, CI95% [1.001–4]; *p* = 0.031). Based on these findings, a severity score was developed incorporating these four factors, each weighted accordingly. The resulting score ranges from ≤2 (with a 5% probability) to ≥40 (with a probability exceeding 99%) for predicting severity (see Table 3). The optimal cut-off value was determined to be 12, demonstrating a sensitivity of 77%, a specificity of 72%, and an overall accuracy of 73%. This scoring system outperformed cryoprecipitate levels alone in predicting severity (see Supplementary Table S1 and Figure S1).

## 4. Discussion

Our comprehensive cohort analysis of 166 patients with cryoglobulinemia provides critical insights into the clinical manifestations and prognostic factors of this complex condition. By comparing subgroups based on cryoprecipitate thresholds, CG type, and disease severity, we unraveled the heterogeneity of the cryoglobulinemia spectrum.

Recognizing that cryoglobulins can be present in asymptomatic patients, we first analyzed the cohort using a cryoprecipitate threshold of 50 mg/L, based on the literature analysis [12,13,14]. Patients with high cryoprecipitate levels were more likely to exhibit constitutional and musculoskeletal symptoms, suggesting a link between cryoprecipitate levels and symptomatology, consistent with existing studies [1,8,22]. Notably, vascular symptoms, hallmark features of the disease, appeared independent of cryoprecipitate levels and correlated instead with the mere presence of cryoglobulins.

This underscores the need for physicians to consider cryoglobulinemia syndrome in patients with vascular symptoms, even with normal complement fractions, echoing the concept of “idiopathic hypo-cryoglobulinemia” proposed by Roccatello et al. [16].

Moreover, the study sheds light on the clinical presentations associated with CG types. Type I and mixed CG represent two different spectra of the same “entity” with different pathophysiology [1,4,6,8]. In the current study, the difference in clinical presentation (splenomegaly) is more related to the underlying conditions, such as lymphoma, rather than the CG type. Patients with type I CG often experience hyperviscosity syndrome or severe vascular complications related to small vessel thrombosis [10]. The limited numbers of type I GC in the study may contribute to the similarities in clinical presentation. Nevertheless, prior large cohort analyses report a worse prognosis associated with type I CG attributed to thrombotic events and association with hematological malignancies impacting long-term survival [10,23]. Additionally, the low prevalence of C-virus hepatitis reflects the evolution of the cryoglobulinemia spectrum in the modern era.

Regarding severity, patients with severe disease exhibited distinctive clinical features including purpura, Raynaud’s phenomenon, and leukocytoclastic vasculitis on skin biopsy. These findings, often associated with type I CG [10,24], likely result from vascular occlusion.

Although severity was subjectively assessed based on physician judgment, the inclusion of cutaneous items from the BVAS 2003 (a scoring system for ANCA-associated vasculitis) lent partial validation to these assessments. Nevertheless, the patients were mainly managed by rheumatologists or internal medicine practitioners, which may represent a bias explaining the low frequency of renal involvement and absence of pulmonary or gastrointestinal manifestations contributing to lowering the overall BVAS scores overall. Given the real-world nature of this retrospective study, treatment initiation, particularly corticosteroid therapy, was used as a proxy for severity staging, though variations in treatment regimens represent a potential source of bias.

Regarding outcomes, the cryoprecipitate level emerged as a key predictor of disease course, influencing relapse-free survival, progression to chronic disease, the need for renal replacement therapy, and one-year overall survival. Neither CG type nor the initial severity predicted these outcomes. However, the relatively short follow-up period limits the ability to assess long-term effects, as mortality related to cryoglobulinemia or its treatments often unfolds over several years [11,25]. Additionally, the absence of precise documentation on causes of death complicates the interpretation of outcomes, making it challenging to isolate cryoglobulinemia’s role from that of comorbidities.

The study’s most innovative contribution is the development of a clinically applicable severity scoring system. Integrating factors identified through multivariate analysis—purpura, hematological conditions, Raynaud’s phenomenon, and elevated cryoprecipitate levels—demonstrated superior predictive accuracy compared to cryoprecipitate levels alone. These tools recapitulate the spectrum of type I CG by incorporating vascular manifestations (i.e., purpura and severe Raynaud’s phenomenon) and underlying hematological conditions [10,23]. Its reliance on readily available clinical and laboratory parameters within 24 h makes it a practical tool for early diagnosis and treatment decision-making. This scoring system not only supports improved patient outcomes but also reinforces the importance of considering cryoglobulinemia syndrome independently of cryoprecipitate thresholds, aligning with previous concepts such as “idiopathic hypo-cryoglobulinemia.” [16].

However, the current study has several limitations. First, its retrospective nature, with potentially missing data and absence of long-term follow-up, is a major pitfall. Secondly, the dosage of cryoprecipitate and the treatment initiation were at the physician’s appreciation based on clinical experience rather than a standardized protocol. Additionally, the use of cryoprecipitate threshold cutoff based on the literature analysis rather than statistical methods warrants caution. Moreover, an external validation is necessary to confirm the score’s reliability.

Nevertheless, the primary strength of the study lies in its ability to provide an overview of the clinical manifestations of cryoglobulinemia syndrome based on cryoprecipitate levels and, notably, in proposing a severity score applicable for clinical use. The proposed severity score is straightforward, clinically useful, and holds promise for enhancing the management of cryoglobulinemia syndrome. Future studies should aim to validate the score and explore its application in multicentric studies.

In conclusion, cryoglobulinemic vasculitis must be considered independently of the cryoprecipitate threshold and type. Moreover, a novel severity scoring system based on four items (purpura, Raynaud’s phenomenon, hematological conditions, and cryoprecipitate) may predict severity and clinical decision-making and patient care.

## Figures and Tables

**Table 1:** Patient characteristics according to cryoglobulins threshold.

Patients Characteristics	Total (n = 166)	Cryo ≤ 50 g/L (n = 102)	Cryo > 50 g/L (n = 64)	*p* Value
Age median [IQR]	66 [53.75–77.0]	65.0 [51–77.25]	67.50 [57–77]	0.160
Sex male n (%)	76 (45%)	49 (48%)	27 (42%)	0.523
Type of cryoglobulinemia				
Cryoglobulin type I	16 (9.6%)	8 (7.84%)	8 (12.5%)	0.4187
Cryoglobulin type II	77 (46.38%)	44 (43.13%)	33 (51.56%)	0.3383
Cryoglobulin type III	60 (36%)	38 (37.25%)	22 (34.37%)	0.7423
Underlying conditions				
Monoclonal gammopathy	9 (5.42%)	4 (3.92%)	5 (7.8%)	0.2795
Waldenström macroglobulinemia	7 (4.21%)	3 (2.94%)	4 (4.68%)	0.2416
Diffuse large B-cell lymphoma	8 (4.18%)	7(6.98%)	1 (1.56%)	0.2930
C-virus hepatitis associated cryoglobulinemia	24 (14.45%)	7 (6.8%)	17 (26%)	0.0007
Rheumatic arthritis	12 (7.2%)	8 (7.84%)	4 (6.25%)	0.9211
SLE	6 (3.61%)	5 (4.90%)	1 (1.56%)	0.4310
Sjogren	6 (3.61%)	5 (4.90%)	1 (1.56%)	0.4310
Constitutional symptoms				
Fatigue	23 (13%)	6 (5%)	17 (26%)	0.001
Fever	16 (9%)	5 (4%)	11 (17%)	0.013
Weigh loss (>10% of BMI in 6 months or 5% one month)	6 (3%)	0 (0%)	6(9%)	0.003
Musculoskeletal symptoms	14 (8%)	3 (2%)	11 (17%)	0.002
Vascular symptoms				
Purpura	29 (17%)	13 (12%)	16 (25%)	0.058
Raynaud’s phenomenon/acrocyanosis	19 (11%)	10 (9%)	9 (14%)	0.457
Livedo reticularis	7 (4%)	3 (2%)	4 (6%)	0.432
Necrosis	10 (6%)	8 (7%)	2 (3%)	0.318
Leukocytoclasic vasculitis on skin biopsy	13/34 (38%)	5/15 (4%)	8/19 (42%)	0.134
Neurological features				
Sensitive deficiency	16 (9.6%)	12 (11.7%)	4 (6.25%)	0.02838
Motor deficiency	11 (6.62%)	5 (4%)	6 (9%)	>0.999
Compatible neurologic features at electromyogram	2 (1%)	1 (0.9%)	1 (1%)	>0.999
Enlarged lymph nodes	9 (5%)	3 (2%)	6 (9%)	0.156
Splenomegaly	13 (7%)	7 (6%)	6 (9%)	0.768
Proteinuria > 0.5 g/day	23/67 (34%)	12/45 (26.6%)	11/22 (50%)	0.0988
Hematuria > 10/champ	28/67 (41.7%)	17/44 (38.6%)	11/23 (47%)	0.6028
Creatinine level (µmol/L)	76.5 [63–102]	75 [59–98]	78.5 [65–117.5]	0.2926
(CH50)	52 [39–76]	64 [46–76]	43 [20–61]	0.017
C3	1.01 [0.762–1.28]	1.08 [0.82–1.35]	0.9 [0.65–1.19]	0.028
C4	0.16 [0.09–0.255]	0.1850 [0.110–0.277]	0.12 [0.045–0.20]	0.006
Outcomes at one year				
Absence of relapse	60 (36%)	28 (27%)	32 (50%)	0.0046
Evolution in chronic disease > 2 relapses	63 (37%)	63 (61%)	0 (0%)	<0.0001
Renal replacement therapy	13 (7.83%)	1 (0.98%)	12 (18%)	<0.0001

BMI: body mass index; SLE: systemic lupus erythematosus.

**Table 2:** Patients characteristics according to cryoglobulinemia type.

Patients Characteristics	Total (n = 166)	Type 1 (n = 16)	Mixed Cryo (n = 150)	*p* Value
Age median [IQR]	66 (53.75–77)	68.5 (64–77)	66 (53–68.50)	0.2952
Sex male n (%)	76 (45.7%)	11 (68.7%)	65 (43%)	0.1998
Underlying conditions before diagnosis				
Hematological malignancies	14 (8.4%)	5 (31%)	9 (6%)	0.0068
C-virus hepatitis	27 (16.2%)	0 (0%)	27 (18%)	0.0761
SLE	5 (3%)	1 (6.25%)	4 (2.6%)	>0.9999
Sjogren		1 (6.25%)	9 (6%)	>0.9999
Underlying conditions at diagnosis				
Hematological malignancy	26 (15.6%)	8 (50%)	18 (12%)	0.0011
Waldenström macroglobulinemia	7 (4.2%)	7 (43%)	0 (0%)	<0.0001
C-virus hepatitis reactivation	16 (9.6%)	0 (0%)	16 (10.6%)	0.2242
Constitutional symptoms				
Fatigue	40 (24%)	2 (12.5%)	38 (25.3%)	0.2362
Fever	15 (3%)	0 (0%)	15 (10%)	0.2230
Weigh loss (>10% of BMI in 6 months or 5% one month)	10 (6%)	2 (12.5%)	8 (5.3%)	0.6016
Musculoskeletal symptoms	25 (15%)	0 (0%)	25 (16.6%)	0.0745
Vascular symptoms				
Purpura	25 (15%)	2 (12.5%)	23 (15.3%)	0.7365
Raynaud’s phenomenon/acrocyanosis	21 (12.6%)	1 (6.25%)	20 (13.3%)	0.4589
Livedo reticularis	13 (7.8%)	1 (6.25%)	12 (8%)	>0.9999
Necrosis	3 (1.8%)	0 (0%)	3 (2%)	>0.9999
Leukocytoclasic vasculitis on skin biopsy	13/29 44(%)	1/2 (50%)	12/27 (8%)	0.665
Neurological features				
Sensitive deficiency	15 (3%)	1 (6.25%)	14 (9.3%)	0.7059
Motor deficiency	7 (4.2%)	0 (0%)	7 (4.6%)	0.6080
Compatible neurologic features at electromyogram	2 (1.2%)	0 (0%)	2 (1.33%)	>0.9999
Enlarged lymph nodes	8 (4.8%)	1 (6.25%)	7 (4.66%)	>0.9999
Splenomegaly	12 (7.2%)	5 (31.25%)	7 (4.66%)	0.0039
Proteinuria > 0.5 g/day	22/65 (33.8%)	1/10 (10%)	21/55 (38%)	0.1449
Hematuria > 10/champ	26/65 (40%)	1/10 (10%)	25/55 (45%)	0.751
Creatinine level (µmol/L)	76.5 (63–102)	76 (65–94)	78 (64–106)	0.5333
(CH50) UI/mL	51 (39–76)	78 (58–193)	51 (32.25–76)	0.0350
C3 g/L	1.10 (0.76–1.29)	1.08 (0.94–1.2)	1.010 (0.75–1.28)	0.5868
C4 g/L	0.16 (0.09–0.26)	0.1450 (0.070)	0.16 (0.0850)	0.3997

BMI: body mass index; SLE: systemic lupus erythematosus.

**Table 3:** Patients characteristics according to CG severity at diagnosis.

Patients Characteristics	Total (n = 166)	Non-Severe (n = 133)	Severe (n = 33)	*p* Value
Age median [IQR]	66 [53.75–77.0]	66 [54.5–77.5]	69.5 [53–77.5]	0.4394
Sex male n (%)	75 (45.1%)	61(45.8%)	14 (42.4%)	>0.9999
Underlying conditions before diagnosis				
Hematological malignancies	15 (9%)	11 (8.2%)	4 (12.1%)	0.1433
C-virus hepatitis	25 (15%)	20 (15%)	5 (15.1%)	>0.999
SLE	6 (3.6%)	6 (4.5%)	0 (0%)	0.3617
Sjogren	11 (6.6%)	8 (6%à	3 (9%)	0.3537
Underlying conditions at diagnosis				
Hematological malignancy		17 (12.7%)	9 (27%)	0.0279
Waldenström macroglobulinemia	43 (26%)	36 (27%)	7 (21%)	0.673
C-virus hepatitis reactivation	17 (10%)	16 (12%)	1 (3%)	0.153
Constitutional symptoms				
Fatigue	43 (25.9%)	36 (27%)	7 (21%)	0.6573
Fever	17 (10.2%)	16 (12%)	1 (3%)	0.1526
Weight loss > 10%	11 (6%)	8 (6%)	3 (9%)	0.691
Musculoskeletal symptoms	27 (16%)	22 (16%)	5 (15%)	0.893
Vascular symptoms				
Purpura	29 (17%)	15 (11%)	14 (42%)	<0.001
Raynaud’s phenomenon/acrocyanosis	22 (13%)	14 (10%)	8 (24%)	0.039
Livedo reticularis	14 (8%)	11 (8%)	3 (9%)	>0.9999
Necrosis	3 (1%)	1 (0.01%)	2 (6%)	0.095
Leukocytoclasic vasculitis on skin biopsy	14 (8%)	4/26 (15%)	10/15 (66%)	<0.0001
Neurological features				
Sensitive deficiency	16 (9.6%)	13 (9.7%)	3 (9%)	>0.999
Motor deficiency	8 (4.8%)	7 (5.2%)	1 (3%)	0.704
Compatible neurologic features at electromyogram	6 (3.6%)	4 (3%)	2 (6%)	0.6203
Enlarged lymph nodes	9 (5.4%)	8 (6%)	1 (3%)	0.6919
splenomegaly	13 (7%)	8 (6%)	5 (15%)	0.132
Proteinuria > 0.5 g/day	24/83 (28.8%)	20/53 (37.7%)	4/30 (13%)	0.0234
Hematuria > 10/champ	28/82 (34%)	23/52 (44%)	5/30 (16%)	0.0152
Creatinine level (µmol/L)	77 [63–104]	76 [61.7–104]	82 (67–102)	0.5827
Total hemolytic complement (CH50) median [IQR]	51 [39–76]	51 [40.5–73.5]	53 [35.76]	0.804
C3 median [IQR]	1.010 [0.7650–1.010]	1.010 [0.7550–1.280]	1.015 [0.7825–1.428]	0.599
C4 median [IQR]	0.160 [0.090–0.260]	0.170 [0.100–0.2500]	0.1250 [0.060–0.3025]	0.402
Positive rheumatoid factor n(%)	36 (22%)	28 (21%)	8 (26%)	0.784
Cryoglobulins dosage	0.0370 [0.0250–0.070]	0.03360 [0.025–0.0650]	0.054 [0.0280–0.1915]	0.010
BVAS SCORE				
Overall	3 [0–6]	3 [0–6]	2 [1–7.25]	0.6531
General signs	0 [0–1]	0 [0–1]	0 [0–1]	0.6914
Skin	0 [0–2]	0 [0–2]	2 [0–6]	0.0047
Neurological	0 [0–0]	0 [0–0]	0 [0–0]	0.8957
Kidney	0 [0–1]	0 [0–0]	0 [0–0]	0.6495

BVAS: Birmingham vasculitis activity score. The score is calculated based on the items with ponderation. The results are presented as a median with interquartile range.

## Data Availability

The original contributions presented in this study are included in the article. Further inquiries can be directed to the corresponding author.

## Supplementary Materials

The following supporting information can be downloaded at: https://www.mdpi.com/article/10.3390/jcm14020556/s1, Figure S1: Area under the receiver operating characteristic curve for the severity score and serum cryoglobulin. Table S1: The severity score.

## References

[1] Ramos-Casals M., Stone J.H., Cid M.C., Bosch X. (2012). The cryoglobulinaemias. Lancet.

[2] Brouet J.-C., Clauvel J.-P., Danon F., Klein M., Seligmann M. (1974). Biologic and clinical significance of cryoglobulins. A report of 86 cases. Am. J. Med..

[3] Meltzer M., Franklin E.C. (1966). Cryoglobulinemia—A study of twenty-nine patients. I. IgG and IgM cryoglobulins and factors affecting cryoprecipitability. Am. J. Med..

[4] Cacoub P., Vieira M., Saadoun D. (2024). Cryoglobulinemia-One Name for Two Diseases. N. Engl. J. Med..

[5] Ramos-Casals M., Trejo O., García-Carrasco M., Cervera R., Font J. (2000). Mixed cryoglobulinemia: New concepts. Lupus.

[6] Desbois A.C., Cacoub P., Saadoun D. (2019). Cryoglobulinemia: An update in 2019. Jt. Bone Spine.

[7] Quartuccio L., Isola M., Corazza L., Ramos-Casals M., Retamozo S., Ragab G.M., Zoheir M.N., El-Menyawi M.A.-M., Salem M.N., Sansonno D. (2014). Validation of the classification criteria for cryoglobulinaemic vasculitis. Rheumatology.

[8] Roccatello D., Saadoun D., Ramos-Casals M., Tzioufas A.G., Fervenza F.C., Cacoub P., Zignego A.L., Ferri C. (2018). Cryoglobulinaemia. Nat. Rev. Dis. Primers.

[9] Terrier B., Karras A., Kahn J.-E., Le Guenno G., Marie I., Benarous L., Lacraz A., Diot E., Hermine O., de Saint-Martin L. (2013). The spectrum of type I cryoglobulinemia vasculitis: New insights based on 64 cases. Medicine.

[10] Ghembaza A., Boleto G., Bommelaer M., Karras A., Javaugue V., Bridoux F., Alyanakian M., Frenkel V.M., Ghillani-Dalbin P., Musset L. (2023). Prognosis and long-term outcomes in type I cryoglobulinemia: A multicenter study of 168 patients. Am. J. Hematol..

[11] Terrier B., Carrat F., Krastinova E., Marie I., Launay D., Lacraz A., Belenotti P., Martin L.d.S., Quemeneur T., Huart A. (2013). Prognostic factors of survival in patients with non-infectious mixed cryoglobulinaemia vasculitis: Data from 242 cases included in the CryoVas survey. Ann. Rheum Dis..

[12] Napodano C., Gulli F., Rapaccini G.L., Marino M., Basile U. (2021). Cryoglobulins: Identification, classification, and novel biomarkers of mysterious proteins. Adv. Clin. Chem..

[13] Gorevic P.D., Kassab H.J., Levo Y., Kohn R., Meltzer M., Prose P., Franklin E.C. (1980). Mixed cryoglobulinemia: Clinical aspects and long-term follow-up of 40 patients. Am. J. Med..

[14] Trendelenburg M., Schifferli J.A. (1998). Cryoglobulins are not essential. Ann. Rheum. Dis..

[15] Ferri C., Zignego A.L., Pileri S.A. (2002). Cryoglobulins. J. Clin. Pathol..

[16] Roccatello D., Sciascia S., Naretto C., Barreca A., Solfietti L., Battaglia L., Viziello L., Fenoglio R., Rossi D. (2022). Recognizing the new disorder “idiopathic hypocryoglobulinaemia” in patients with previously unidentified clinical conditions. Sci. Rep..

[17] Terrier B., Darbon R., Durel C.-A., Hachulla E., Karras A., Maillard H., Papo T., Puechal X., Pugnet G., Quemeneur T. (2020). French recommendations for the management of systemic necrotizing vasculitides (polyarteritis nodosa and ANCA-associated vasculitides). Orphanet J. Rare Dis..

[18] Fabrizi F., Plaisier E., Saadoun D., Martin P., Messa P., Cacoub P. (2013). Hepatitis C virus infection, mixed cryoglobulinemia, and kidney disease. Am. J. Kidney Dis..

[19] Ferri C., Cacoub P., Mazzaro C., Roccatello D., Scaini P., Sebastiani M., Tavoni A., Zignego A., De Vita S. (2011). Treatment with rituximab in patients with mixed cryoglobulinemia syndrome: Results of multicenter cohort study and review of the literature. Autoimmun. Rev..

[20] Roccatello D., Sciascia S., Baldovino S., Rossi D., Alpa M., Naretto C., Di Simone D., Menegatti E. (2016). Improved (4 Plus 2) Rituximab Protocol for Severe Cases of Mixed Cryoglobulinemia: A 6-Year Observational Study. Am. J. Nephrol..

[21] Musset L., Diemert M.C., Taibi F., Du L.T.H., Cacoub P., Leger J.M., Boissy G., Gaillard O., Galli J. (1992). Characterization of cryoglobulins by immunoblotting. Clin. Chem..

[22] Terrier B., Marie I., Launay D., Lacraz A., Belenotti P., de Saint-Martin L., Quemeneur T., Huart A., Bonnet F., Le Guenno G. (2014). Predictors of early relapse in patients with non-infectious mixed cryoglobulinemia vasculitis: Results from the French nationwide CryoVas survey. Autoimmun. Rev..

[23] Sidana S., Rajkumar S.V., Dispenzieri A., Lacy M.Q., Gertz M.A., Buadi F.K., Hayman S.R., Dingli D., Kapoor P., Gonsalves W.I. (2017). Clinical presentation and outcomes of patients with type 1 monoclonal cryoglobulinemia. Am. J. Hematol..

[24] Ramos-Casals M., Robles A., Brito-Zerón P., Nardi N., Nicolás J.M., Forns X., Plaza J., Yagüe J., Sánchez-Tapias J.M., Font J. (2006). Life-threatening cryoglobulinemia: Clinical and immunological characterization of 29 cases. Semin. Arthritis Rheum..

[25] Fayed A., Hegazy M.T., Biard L., Vieira M., El Shabony T., Saadoun D., Casato M., Visentini M., Ragab G., Cacoub P. (2022). Relapse of Hepatitis C Virus Cryoglobulinemic Vasculitis After Sustained Viral Response After Interferon-Free Direct-Acting Antivirals. Am. J. Gastroenterol..

