# Supplementary material for: Identification of Key Predictors of Cryoglobulinemia Severity at Diagnosis: Threshold, Type, and Severity Score at Diagnosis
Source: J Clin Med. 2025 Jan 16;14(2):556. doi: 10.3390/jcm14020556 (PMC11765962; doi:10.3390/jcm14020556)
Supplement: Supplementary file 1 [file jcm-14-00556-s001.zip › jcm-3389004-supplementary.pdf]

Figure 1. Area under the receiver operating characteristic curve for the severity score and serum cryoglobulin.
the best cutoff value for the severity score was 12, corresponding to a sensitivity of 77%, a specificity of 72%, and accurate classification of 73% of the patients.

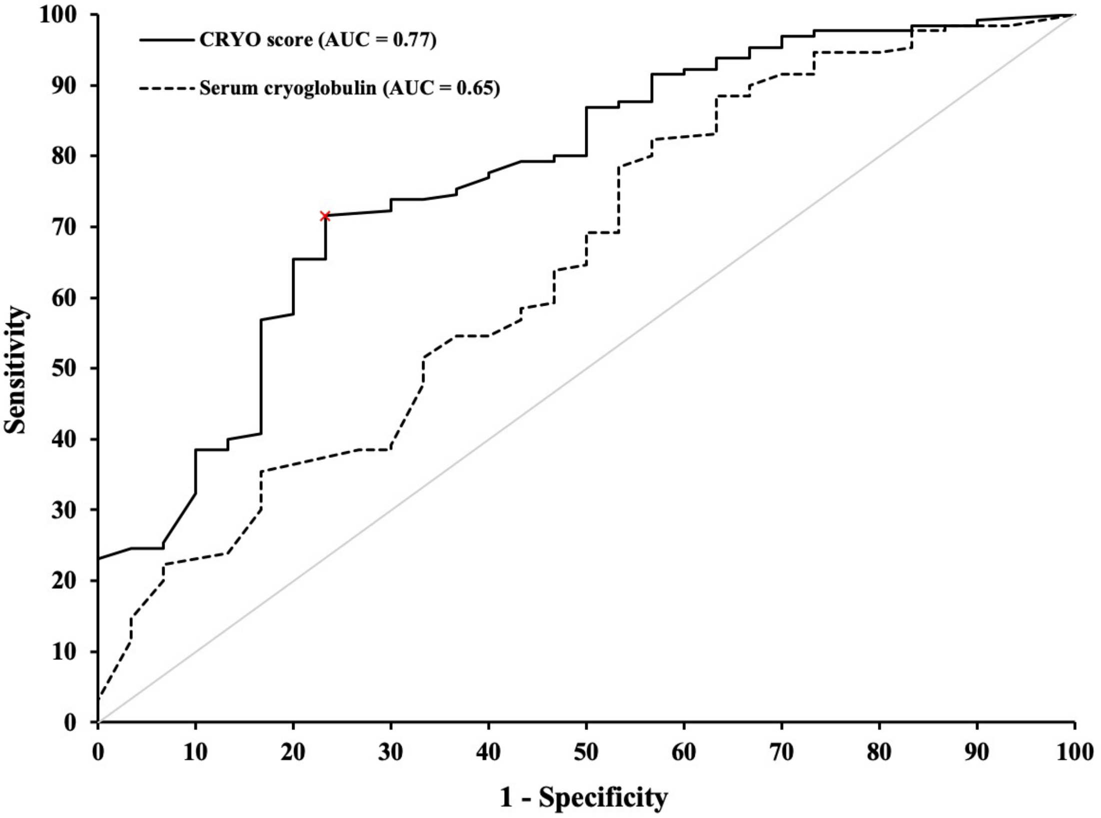

Table S1:

The severity score	
Parameter	No. of points (criteria for scoring)
Serum cryoglobulin, µg/L	Divide serum cryoglobulin per 10
History of hematological disorder	0 (no) or 9 (yes)
Raynaud phenomenon	0 (no) or 9 (yes)
Purpura	0 (no) or 11 (yes)

Probability of severe cryoglobulinemia according to the severity score

CRYO score	Probability (%)
≤ 2	5
4	7.5
6	10
9	20
12	30
14	40
16	50

18	60
20	70
22	80
25	87
30	95
≥ 40	> 99
